# Ex Vivo Immunomodulatory Effects of *Lactobacillus*-, *Lacticaseibacillus*-, and *Bifidobacterium*-Containing Synbiotics on Human Peripheral Blood Mononuclear Cells and Monocyte-Derived Dendritic Cells in the Context of Grass Pollen Allergy

**DOI:** 10.1007/s12602-022-09920-w

**Published:** 2022-02-03

**Authors:** Alexander Heldner, Matthew D. Heath, Benjamin Schnautz, Sebastian Kotz, Adam Chaker, Matthias F. Kramer, Constanze A. Jakwerth, Ulrich M. Zissler, Carsten B. Schmidt-Weber, Simon Blank

**Affiliations:** 1 Center of Allergy and Environment (ZAUM)Faculty of Medicine and Helmholtz Center MunichMember of the German Center of Lung Research (DZL), Member of the Immunology and Inflammation Initiative of the Helmholtz Association, Technical University of Munich, German Research Center for Environmental Health, Ingolstädter Landstraße 1, 85764 Munich, Germany; 2 Allergy Therapeutics PLC, Worthing, UK; 3 Faculty of Medicine, Department of Otolaryngology, Klinikum Rechts Der Isar, Technical University of Munich, Munich, Germany; 4 Bencard Allergie GmbH, Munich, Germany

**Keywords:** *Bifidobacterium*, Grass pollen allergy, Immunomodulation, *Lactobacillus*, *Lacticaseibacillus*, Synbiotics

## Abstract

Sensing of the intestinal microbiota by the host immune system is important to induce protective immune responses. Hence, modification of the gut microbiota might be able to prevent or treat allergies, mediated by proinflammatory Th2 immune responses. The aim was to investigate the ex vivo immunomodulatory effects of the synbiotics Pollagen® and Kallergen®, containing the probiotic bacterial strains *Lactobacillus*, *Lacticaseibacillus* and *Bifidobacterium*, in the context of grass pollen allergy. Peripheral blood mononuclear cells (PBMCs) from grass pollen–allergic patients and healthy controls were stimulated with grass pollen extract (GPE) and synbiotics and Gata3 expression and cytokine secretion analyzed. Monocyte-derived dendritic cells (MoDCs) cells were matured in the presence of GPE and synbiotics, co-cultured with autologous naïve T cells and maturation markers and cytokine secretion analyzed. GPE stimulation of PBMCs from grass pollen–allergic patients resulted in a significant higher production of the Th2 cytokines IL-4, IL-5, IL-9 and IL-13 compared to healthy controls. Gata3^+^CD4^+^ T cell induction was independent of the allergic status. The synbiotics promoted IL-10 and IFN-γ secretion and downregulated the GPE-induced Th2-like phenotype. Co-culturing naïve T cells with MoDCs, matured in the presence of GPE and synbiotics, shifted the GPE-induced Th2 cytokine release towards Th1-Th17-promoting conditions in allergic subjects. The investigated synbiotics are effective in downregulating the GPE-induced Th2 immune response in PBMCs from grass pollen–allergic patients as well as in autologous MoDC-T cell stimulation assays. In addition to increased IL-10 release, the data indicates a shift from a Th2- to a more Th1- and Th17-like phenotype.

**Supplementary Information:**

The online version contains supplementary material available at 10.1007/s12602-022-09920-w.

## Introduction

Respiratory allergies to airborne allergen sources like pollen are the most frequent type-1 hypersensitivities. In developed countries, sensitization rates to grass (*Pooideae*) pollen such as timothy grass (*Phleum pretense*) substantially increased over the last decades and nowadays range between 10 to 30% in the general population [1]. Although pollen-monitoring networks were implemented in the last few years [2], avoidance of exposure to airborne allergens is nearly impossible. Allergen-specific immunotherapy (AIT) is the only curative treatment that is able to improve allergy-driven symptoms by inducing allergen-specific immune tolerance.

Within the last few years novel therapeutic and preventive approaches were investigated. Here, modifying the patients’ gut microbiota with probiotic and prebiotic supplements, known as synbiotic preparations, got into the focus of allergy research, prevention, and treatment [3]. Probiotics are living strains of microorganisms that have beneficial effects on the gut microbiome when administrated in adequate amounts. Prebiotics are non-digestible food ingredients that contribute to the growth and activity of beneficial bacterial strains and resident microorganisms in the gut [4]. It is thought that pro- and prebiotics are able to induce a more favorable gut colonization and strengthen immune function by immunomodulatory properties, thus contributing to the reduction of allergic symptoms as well as of the risk of allergy development in the context of allergy prevention [5, 6]. The modulation mechanisms of probiotic bacteria are highly complex, involving a variety of effector signals, cell types, different receptors, and bacteria strains differing in their ability to trigger these effects. Besides direct affecting the host epithelial barrier function via microorganism‐associated molecular patterns and pattern recognition receptors, these beneficial effects are mediated by modulation of innate immunity by influencing dendritic cell maturation. Shifting the quality of the innate immune response can lead to an increased Th1/Th2 ratio and elevated amount of regulatory T cells (Tregs) [7]. Further studies indicated that probiotic bacteria are acting as Th1/Treg inducers and may be potential candidates as adjuvants in AIT [8, 9]. It is known that an adequate maturation of the intestinal microenvironment and gut colonization in early life is crucial for protection against allergic diseases and maintaining health in adulthood [10]. Several different intestinal microbial strains and their gut colonization in early life are considered to be essential for future health [11]. After birth, fecal microbiota of neonates is dominated by *Prevotella* and *Lactobacillus* strains, then within the first weeks of life actinobacteria, mainly *Bifidobacterium* stains, become more dominant [12, 13]. Several studies showed that breast-fed infants have more Bifidobacteria and lactobacilli and a more diverse intestinal microbial flora [12]. It is known that dysbiosis, characterized by a depletion of certain bacterial strains and altered metabolic activity, especially in childhood, is associated with an increased risk of atopy and asthma development [14]. Immunomodulatory effects of synbiotic preparations, consisting of probiotic lactic acid bacteria strains (e.g., *Lactobacillus* and *Bifidobacterium*) and prebiotic fructo-oligosaccharides (FOS), were recently studied in the context of respiratory allergies such as allergic rhinitis and asthma [15–17]. In addition, in ovalbumin-(OVA-)sensitized mice, probiotic strains were able to reduce OVA-specific IgE (sIgE) levels and airway hyper-responsiveness and, furthermore, to decrease the number of infiltrating inflammatory cells and the levels of Th2 cytokines in bronchoalveolar lavage fluid and serum [18]. Nevertheless, the results are inconclusive so far, and the efficacy of probiotics and prebiotics as potential allergy‐preventive supplements has to be investigated further [19]. Hence, the aim of this study was to gain insights in the ex vivo immunomodulatory capacity of two synbiotic mixes - Pollagen® and Kallergen® - in the context of grass pollen allergy–mediated Th2-type inflammation.

## Methods

### Patients

Peripheral blood samples of patients with a confirmed history of grass pollen allergy and of non-grass pollen–allergic volunteers were used to isolate peripheral blood mononuclear cells (PBMCs), monocytes, and naïve T cells. The diagnosis of grass pollen allergy was based on a combination of clinical history and positive skin prick test and/or positive sIgE level to grass pollen extract (grass pollen extract and rPhl p1/5 (UniCAP250; Thermo Fisher Scientific, Uppsala, Sweden). Healthy volunteers were defined by a lack of allergic symptoms in their patient history and negative skin prick test results. The study was approved by the local ethics committee of the Faculty of Medicine of the Technical University of Munich (299/15 s), and all patients and volunteers gave written informed consent prior to study participation.

### Synbiotic Mixes

Two commercially available synbiotic dietary supplements based on probiotics and prebiotics were tested. Pollagen® (Bencard Allergie GmbH, Munich, Germany) contains *Lactobacillus acidophilus* NCFM (33 billion CFU/100 g), *Bifidobacterium lactis* BL-04 (100 billion CFU/1006), and fructooligosaccharides (FOS) (33.33 g/100 g), and Kallergen® (Bencard Allergie GmbH) contains *Lacticaseibacillus rhamnosus* (previously *Lactobacillus rhamnosus*) LR05 (38.5 billion CFU/100 g), *Bifidobacterium lactis* BS01 (38.5 billion CFU/100 g), and FOS (85.5 g/100 g). The number of colony-forming units (CFU) of both synbiotic mixes was adjusted to the same amount of probiotic bacterial cells in each experimental setup. To obtain synbiotic culture supernatants, 1 × 10^6^ CFU of each synbiotic mix were cultured in 150 ml RPMI medium (Thermo Fisher Scientific, Waltham, Massachusetts, USA) at 37 °C starting at an OD_600_ = 0.1 until a OD_600_ = 0.8 was reached. After centrifugation (3000 rcf, 15 min), culture supernatants were sterile filtered (0.22 µm, Millex-GP filter unit, Merck, Darmstadt, Germany) and stored at 4 °C. Concentrations of probiotic bacteria and supernatant volumes applied in the following experiments were based on their inhibitory capacity on GPE-induced Gata3 expression. In preliminary experiments, different concentrations of the bacteria and different volumes of the supernatants were applied to GPE-stimulated PBMCs, and the volumes with the strongest inhibitory capacity on Gata3 expression while maintaining cell proliferation and viability were selected for further experiments.

### Ex Vivo Stimulation of PBMCs

PBMCs were isolated from peripheral blood of ten healthy and ten timothy grass pollen allergic patients by density gradient centrifugation (Lymphoprep, Stemcell Technologies, Vancouver, Canada) according to the manufacturers’ protocol and seeded into 96-well flat-bottom plates (Nunc, Thermo Fisher Scientific) at a density of 2 × 10^5^ cells per well in 200 µl T cell medium (RPMI, 10% FCS, 1% glutamine, 1% penicillin/streptomycin, 1% non-essential amino acids, 1% Na-pyruvat (all from Thermo Fisher Scientific)) with 5% autologous serum. PBMCs were treated with 10 µg/mL grass pollen extract (GPE, B2-IHRP-13, Allergy Therapeutics Ltd, Worthing, United Kingdom), a pollen extract mixture from 12 temperate zone grasses, and incubated at 37 °C and 5% CO_2_ for 7 days. Furthermore, 2 × 10^5^ CFU/mL of Pollagen® or Kallergen®, w/o 10 µg/mL LPS-RS Ultrapure as TLR4 antagonist (InvivoGen, San Diego, California, USA) and 1 µl Pollagen® or 10 µl Kallergen® supernatant were added. Moreover, the same conditions were tested with the addition of 10 µg/mL TLR2 antibody (PAb-hTLR2, InvivoGen) for selected subjects. A total of 10 µg/mL phytohemagglutinin (PHA, InvivoGen) was added as positive control for PBMC stimulation. Untreated PBMCs were used as negative control. Expression of T-cell surface markers CD3 (APC-conjugated anti-human CD3, BioLegend, San Diego, California, USA), CD4 (AF700-conjugated anti-human CD4, BioLegend), CD45 (BV711-conjugated anti-human CD45, BioLegend), CD25 (BV605-conjugated anti-human CD25, BioLegend), transcription factors Gata3 (AF488-conjugated anti-human Gata3, eBioscience, San Diego, California, USA), and FoxP3 (PerCP/Cy5.5-conjugated ant-human FoxP3, eBioscience) as well as cell viability and proliferation (Zombie NIR™ Fixable Viability Kit, BioLegend; CellTrace™ CFSE Cell Proliferation Kit, Invitrogen, Carlsbad, California, USA) were measured by flow cytometry. FlowJo_V10 software (FlowJo, LLC, Ashland, Oregon, USA) was used for analysis, and gating was done with fluorescence minus one controls (FMOs) and single stains for each subject (Fig. S1). Furthermore, cytokine concentrations in cell culture supernatants were analyzed by LEGENDplex™ Human Th Panel (13-plex) assay (BioLegend) according to the manufacturer’s protocol.

### Generation of Monocyte-Derived Dendritic Cells

PBMCs were isolated from peripheral blood of three grass pollen–allergic patients and three healthy controls as described above, and CD14^+^ monocytes were isolated by magnetic cell separation (MACS, with CD14^+^ beads, Miltenyi Biotec, Bergisch Gladbach, Germany) and analyzed by FACS (living CD14^+^ cells with a purity > 95%, BioLegend Zombie Aqua™ Fixable Viability Kit). For the generation of monocyte-derived dendritic cells (MoDCs) 3 × 10^6^ CD14^+^ monocytes were seeded into 6-well flat-bottom cell culture plates (Falcon, Thermo Fisher Scientific) in 3 mL T cell medium supplemented with 50 U/mL rhIL-4/rhGM-CSF (both from Miltenyi Biotec) and incubated at 37 °C and 5% CO_2_ for 7 days. At day 4, the same amount of T cell medium supplemented with 50 U/mL rhIL-4/rhGM-CSF was added. After 7 days, immature MoDC (the purity of CD14^−^ MoDCs was > 99% (data not shown)) was analyzed by FACS analysis (APC-Cy7-conjugated anti-human CD80, PE-CF594-conjugated anti-human CD86, FITC-conjugated anti-human CD209, PerCP-Cy5.5-conjugated anti-human HLA-DR, and AF700-conjugated anti-human CD14, all from BD Biosciences, San Jose, CA, USA), and viability was checked by propidium iodide staining.

### Stimulation and Maturation of MoDCs

A total of 1 × 10^5^ cells/mL immature MoDCs were seeded into 96-well flat-bottom plates (Nunc, Thermo Fisher Scientific) and stimulated with 1 µg/mL lipopolysaccharides (LPS) or 1 µg/mL lipoteichoic acid (LTA) as positive control or 1 µg/mL GPE w/o 2 × 10^5^ CFU/mL Pollagen®, 1 µl Pollagen® supernatant, 2 × 10^5^ CFU/mL Kallergen®, or 10 µl Kallergen®. Untreated MoDCs were used as negative control. After 24 h of maturation at 37 °C and 5% CO_2_, cytokine concentrations in culture supernatants were analyzed by LEGENDplex™ Human Inflammation Panel 1 (13-plex) assay (BioLegend) according to the manufacturer’s protocol. Furthermore, the expression of maturation markers CD80 (APC-Cy7-conjugated anti-human CD80, BD Biosciences), CD83 (APC-conjugated anti-human, BD Biosciences), CD86 (PE-CF594-conjugated anti-human CD86, BD Biosciences), CD209 (FITC-conjugated anti-human CD209, BD Biosciences), HLA-DR (PerCP-Cy5.5-conjugated anti-human HLA-DR, BD Biosciences), CD14 (AF700-conjugated anti-human CD14, BD Biosciences), and viability (Zombie NIR™ Fixable Viability Kit, BioLegend) was analyzed by flow cytometry.

### Co-culturing of Mature MoDCs and Naïve T Cells

For autologous stimulation assays, the matured MoDCs were washed and co-incubated with naïve T cells at a ratio of 1:10 in T cell medium at 37 °C and 5% CO_2_ for 7 days. Naïve T cells were isolated from PBMCs using the MACS naïve CD4^+^ T Cell Isolation Kit II (Miltenyi Biotec). A total of 20 U/mL rhIL-2 (Preprotech, Rocky Hill, New Jersey, USA) were added to the cells on the third day. After 7 days, cytokine concentrations in cell culture supernatants were analyzed using the LEGENDplex™ Human Th Panel (13-plex) assay (BioLegend) according to the manufacturer’s protocol.

### Statistical Analyses

Differences between patient groups and stimulation conditions were analyzed by two-way analysis of variance (ANOVA) (GraphPad Prism, San Diego, California, USA). *P*‐values of ≤ 0.05, ≤ 0.01, ≤ 0.001, and ≤ 0.0001 are shown as *, **, ***, and ****, respectively.

## Results

### The GPE-Induced Th2-Type Response in Allergic Patients’ PBMCs is Downregulated by Synbiotic Mixes

**Fig. 1:**
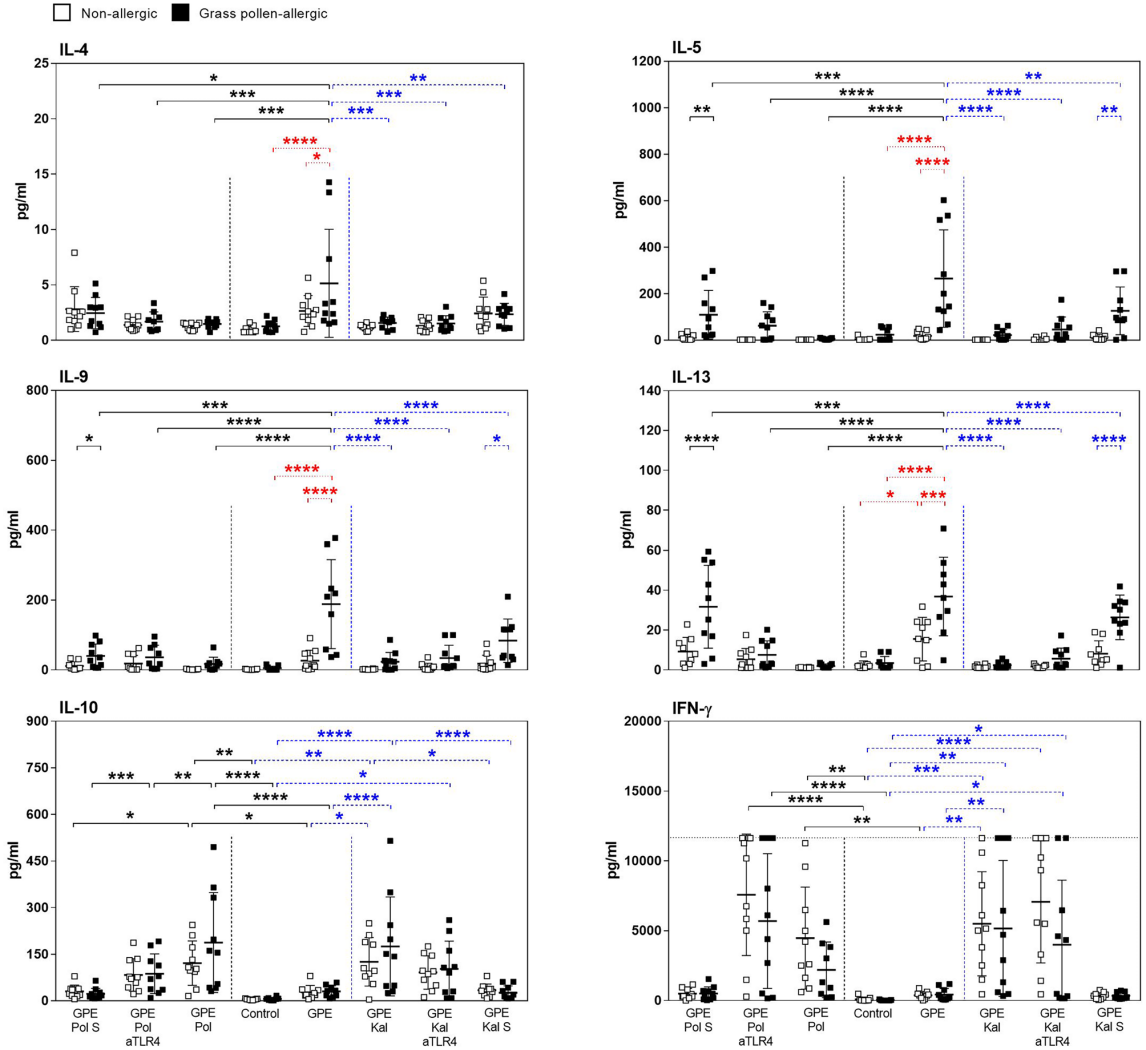
Downregulation of grass pollen extract-driven Th2 cytokine production in grass pollen–allergic patients’ PBMCs by synbiotic mixes. PBMCs from grass pollen–allergic patients (*n* = 10; black squares) and healthy controls (*n* = 10; white squares) were stimulated with grass pollen extract (GPE). Stimulation was done w/o Pollagen (Pol)/Kallergen (Kal), LPS-RS Ultrapure (aTLR4), and Pollagen/Kallergen supernatant (Pol S / Kal S). Supernatants were analyzed for the levels of IL-4, IL-5, IL-9, IL-13, IL-10, and IFN-γ. A dotted line indicates the upper detection limit for IFN-γ. Results are presented as mean ± SD. Significant differences between controls and Pollagen, controls and Kallergen and within controls are indicated in black, blue, and red, respectively

**Fig. 2:**
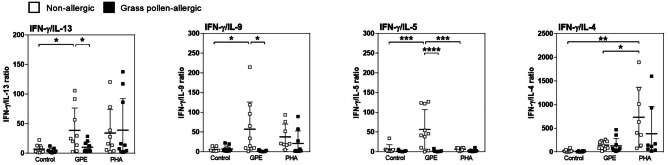
Ratio of Th1 and Th2 cytokines in culture supernatants of grass pollen extract-stimulated PBMCs. PBMCs from grass pollen–allergic patients (*n* = 10; black squares) and healthy controls (*n* = 10; white squares) were stimulated with grass pollen extract (GPE) for 7 days. Phytohemagglutinin (PHA) served as positive control. Supernatants were analyzed for the levels of IL-4, IL-5, IL-9, IL-13, and IFN-g and ratios calculated. Results are presented as mean ± SD

**Fig. 3:**
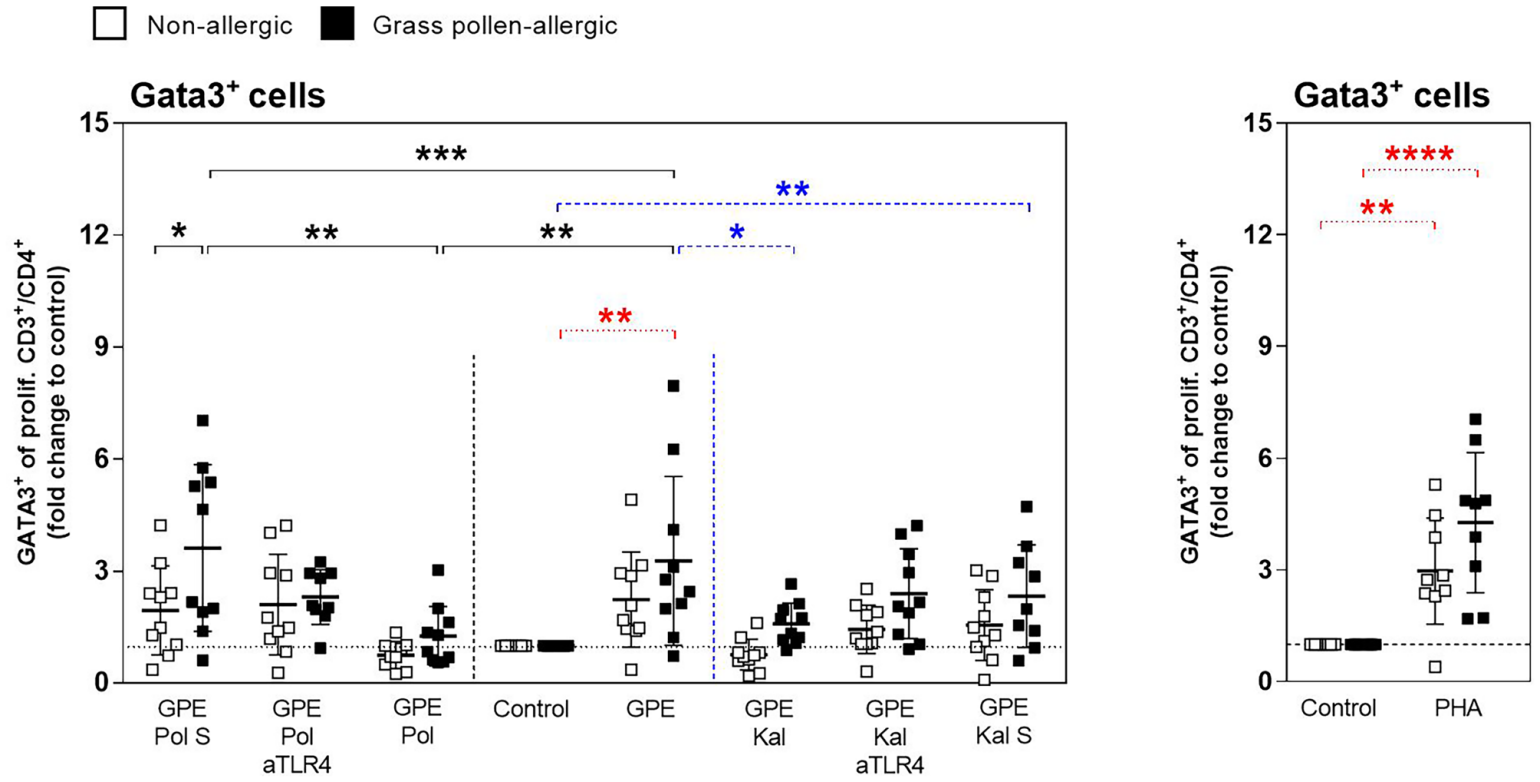
Downregulation of grass pollen extract–induced Gata3 expression in PBMCs by synbiotic mixes. PBMCs from grass pollen–allergic patients (*n* = 10; black squares) and healthy controls (*n* = 10; white squares) were stimulated with grass pollen extract (GPE) for 7 days. Stimulation was done w/o Pollagen (Pol)/Kallergen (Kal), LPS-RS Ultrapure (aTLR4), and Pollagen/Kallergen supernatant (Pol S/Kal S). Phytohemagglutinin (PHA) served as positive control. Gata3^+^ cells of proliferating CD3^+^/CD4^+^ cells were analyzed by FACS. For each patient, Gata3 expression in the negative control was set as 1, and fold change to control was calculated for each stimulation condition. Results are presented as mean ± SD. Significant differences between controls and Pollagen, controls and Kallergen, and within controls are indicated in black, blue, and red, respectively

First, the effects of GPE, synbiotic mixes, and their sterile filtered culture supernatants, containing prebiotics and probiotic metabolites, on PBMCs of grass pollen–allergic patients and healthy controls were analyzed. Stimulation of PBMCs from allergic patients with GPE resulted in a significant production of the Th2 cytokines IL-4, IL-5, IL-9, and IL-13. In healthy controls, only IL-13 was significantly upregulated upon stimulation with GPE. In contrast, in both groups, GPE induced neither IL-10 nor IFN-γ release (Fig. 1). Stimulation with the lymphocyte-stimulating mitogen PHA served as positive control and resulted in cytokine secretion independent of the allergic status of the subjects (Fig. S2). The GPE-induced release of all Th2 cytokines was downregulated by co-culturing with both investigated synbiotic mixes, Pollagen® and Kallergen®. Furthermore, both synbiotic mixes induced IL-10 and IFN-γ release independent of the allergic status (Fig. 1). In healthy controls, stimulation with GPE led to a Th1-skewed response as demonstrated by the ratio of IFN-γ to Th2 cytokine levels (Fig. 2). IL-17A and IL-17F are upregulated upon GPE stimulation and downregulated by addition of the synbiotic mixes in both groups (Fig. S3). Synbiotic culture supernatants were less able to downregulate the GPE-induced modulation of cytokine production and also showed no effect on IL-10 and IFN-γ production compared to direct co-culturing with both synbiotic mixes (Fig. 1). In contrast to Th2 cytokine release, GPE-induced Gata3 expression in proliferating CD4^+^ T cells showed a significant upregulation in allergic subjects as well as an increased trend in non-allergic subjects. This effect was reversed by addition of both synbiotic mixes (Fig. 3). Addition of the TLR4 antagonist LPS-RS Ultrapure as well as of a TLR2 antibody partially blocked the effects of the synbiotic mixes (Figs. 1 and S4). In contrast to Gata-3, GPE-induced expression of FoxP3 in proliferating CD4^+^ T cells, which was higher in allergic patients compared to healthy controls, was not influenced by the addition of the synbiotic mixes (Fig. S5).

### Synbiotic Mixes Induce IL-10 and IL-12p70 Secretion of GPE-Matured MoDCs

**Fig. 4:**
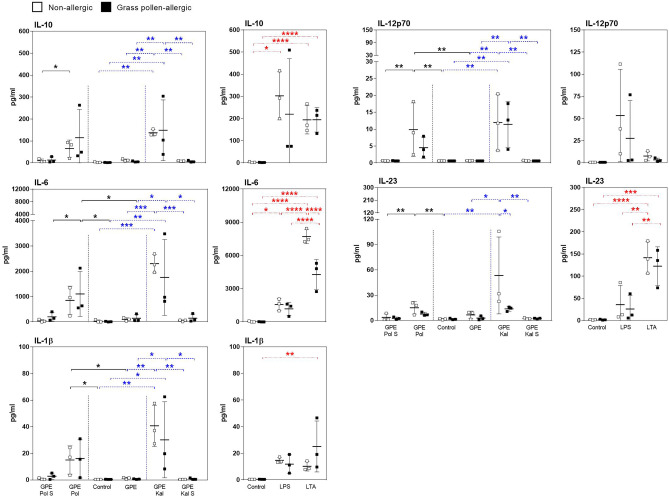
Cytokine secretion of matured MoDCs. Immature MoDCs from grass pollen–allergic patients (*n* = 3; black squares) and healthy controls (*n* = 3; white squares) were stimulated with grass pollen extract (GPE) for 24 h. Stimulation was done w/o Pollagen (Pol)/Kallergen (Kal) and Pollagen/Kallergen supernatant (Pol S/Kal S). Lipopolysaccharide (LPS) and lipoteichoic acid (LTA) served as positive controls. Supernatants were analyzed for the levels of IL-10, IL-12p70, IL-6, IL-23, and IL-1β. Results are presented as mean ± SD. Significant differences between controls and Pollagen, controls, and Kallergen and within controls are indicated in black, blue, and red, respectively

To examine how synbiotics can shift the cytokine secretion of GPE-matured MoDCs, immature MoDCs were stimulated with GPE in the presence of synbiotics or their culture supernatants. All maturation conditions led to an upregulation of the DC surface maturation markers CD83, CD86, and HLA-DR (Fig. S6). Stimulation with LPS and LTA served as positive control (Fig. S6). While maturation in the presence of GPE did not result in significant upregulation of IL-10, IL-12p70, IL-6, IL-23, and IL-1β secretion into culture supernatants, the addition of both synbiotic mixes enhanced production of all five cytokines independent of the allergic status of the studied subjects. This was not the case when MoDCs were matured in the presence of bacterial culture supernatants (Fig. 4). Moreover, there was a tendency toward higher IL-5 and IL-13 secretion from GPE-matured MoDCs of allergic patients (Fig. S7).

### Synbiotic Mixes Shift the GPE-Induced Th2 Cytokine Profile in Autologous Co-cultures of Naïve T Cells and Matured MoDCs to a More Th1- and Th17-Promoting Milieu

**Fig. 5:**
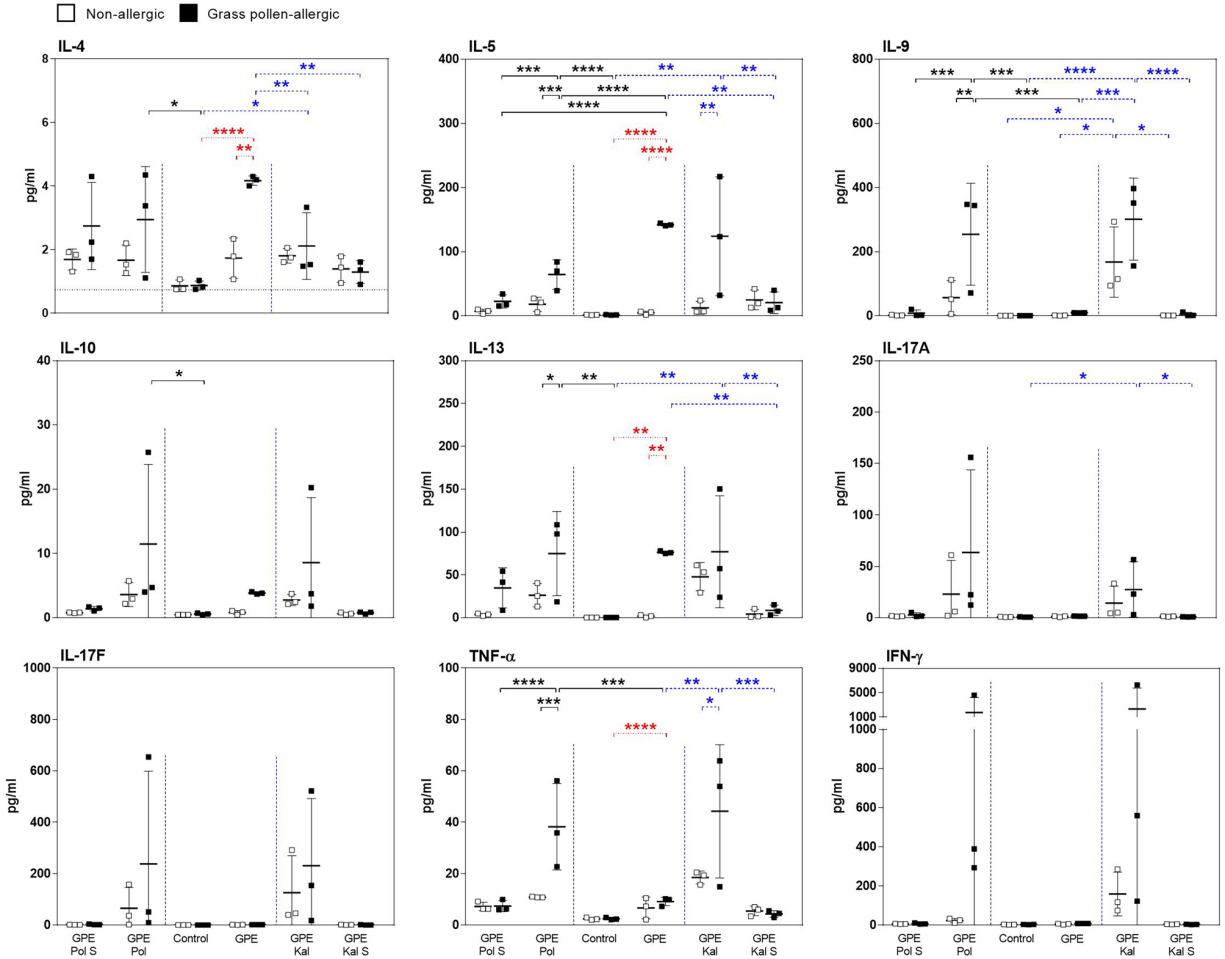
Synbiotic mixes shift the grass pollen extract-(GPE)–induced Th2 cytokine profile in autologous co-cultures of naïve T cells and matured MoDCs to a more Th1- and Th17-promoting milieu. Autologous naïve T cells from grass pollen–allergic patients (*n* = 3; black squares) and healthy controls (*n* = 3; white squares) were co-cultured with GPE w/o Pollagen (Pol)/Kallergen-(Kal) or Pollagen/Kallergen supernatant (Pol S/Kal S) matured MoDCs from the same donors. Supernatants were analyzed for the levels of IL-4, IL-5, IL-13, IL-10, Il-17A, IL-17F, IFN-γ, and TNF-α. Results are presented as mean ± SD. Significant differences between controls and Pollagen, controls and Kallergen, and within controls are indicated in black, blue, and red, respectively

Matured MoDCs were co-cultured with autologous naïve T cells and cytokine profiles analyzed in culture supernatants. Significant higher levels of the Th2 cytokines IL-4, IL-5, and IL-13 were detected in co-cultures of naïve T cells and GPE-matured MoDCs from grass pollen–allergic patients. Although the results showed variability within the individual patients, Pollagen® and Kallergen® were able to significantly decrease the levels of IL-5 and IL-4, respectively (Fig. 5). Intriguingly, this observation was not true for IL-9, which was upregulated only in co-cultures that contained MoDCs that were matured in the presence of GPE and synbiotic mixes, independent of the allergic status (Fig. 5). Interestingly, the synbiotic culture supernatants resulted in comparable or even more suppressive effects on IL-4, IL-5, and IL-13. Moreover, synbiotic mixes resulted in higher levels of IL-17A, IL-17F, IFN-γ, and TNF-α in co-culture supernatants. In the case of IFN-γ and TNF-α, this effect was more pronounced for grass pollen–allergic patients. Both synbiotics had only minimal effects on IL-10 levels (Fig. 5). Controls with LPS- and LTA-matured MoDCs are shown in Fig. S8.

## Discussion

In the first part of the study, the ex vivo response of PBMCs from grass pollen–allergic patients and healthy controls to GPE alone and in the additional presence of the two synbiotic mixes Pollagen® (*Lactobacillus acidophilus* NCFM, *Bifidobacterium lactis* BL-04, FOS) and Kallergen® (*Lacticaseibacillus rhamnosus* LR05, *Bifidobacterium lactis* BS01, FOS) was investigated. In fact, this study does not allow the precise contribution of each individual probiotic strain to the observed effects. However, since the immunological effects of individual strains can differ greatly from mixtures of these strains [20], the detailed study of synbiotic mixes that are available for application in humans is of high interest. Hence, this study aims to provide important evidence for the evaluation if this unique combination of the synbiotics. The relatively low-quality evidence, limited comparative studies, and large heterogeneity between studies have hampered recommendations on specific probiotic formulations for the most effective management. As such, this research is needed piece-by-piece to support the development of specific practical guidelines for dietary supplements containing probiotics and prebiotics.

Stimulation with GPE induced a comparable upregulation of Gata3 in proliferating CD4^+^ T cells independent of the allergic status of the subjects. According to other studies, GPE induced a higher Th2 cytokine release (IL-4, IL-5, IL-9, and IL-13) from PBMCs of allergic patients compared to healthy controls [21, 22]. It was proposed that Gata3 not directly regulates IL-4 but rather acts as a chromatin-remodeling factor, to make the IL-4/IL-5 locus accessible by interacting with other regulatory factors [23, 24]. The GPE-induced Th2 cytokine secretion was completely abolished by co-culturing the stimulated PBMCs with the synbiotic mixes while the addition of synbiotic culture supernatants was less effective. In all groups, the presence of probiotic bacteria seemed to induce a shift to a more Th1-like phenotype as indicated by the increased IFN-γ levels in the culture supernatants. Furthermore, both synbiotic mixes significantly increased IL-10 secretion, which plays a central role in inflammation control by suppressing the release of pro-inflammatory cytokines [25]. The synbiotic mixes did not affect GPE-induced FoxP3 expression in proliferating CD4^+^ T cells, indicating that a quantitative shift in this T cell population with a potentially regulatory phenotype was not responsible for the changes observed at the cytokine level. Taken together, in this study, it could be demonstrated that GPE, which induces a Th2-like response in grass pollen–allergic patient, led to a Th1-skewed response in healthy controls as indicated by an increased ratio of secreted IFN-γ to Th2 cytokines like IL-13, IL-9, IL-5, and IL-4. Overall, the increased Th1/Th2 cytokine ratios are higher in healthy subjects compared to grass pollen–allergic patients. The observed Th1-skewed response in healthy controls was also reported in context of a peanut allergy study [26].

Furthermore, this study investigated the involvement of TLRs in the immunomodulatory effects of the synbiotic mixes. TLRs are membrane-bound pattern recognition receptors (PRRs) which play a crucial role in innate immune responses and are expressed on immune cells, such as dendritic cells, monocytes, and epithelial cells. TLRs on host cells are essential for the recognition of conserved pathogen-associated molecular patterns (PAMPs) of microbial strains and pathogens. Signaling is followed by diverse and tightly regulated mechanisms which mediate the initiation of adaptive immune responses and release of cytokines. Blocking TLR4 as well as TLR2 signaling partially weakened the synbiotics-mediated reduction of the GPE-induced Th2 polarization in PBMCs from allergic patients. These results implicate that the immunomodulatory effects are partially mediated by TLR4 as well as TLR2. In general, lipoteichoic acid of gram-positive probiotic bacteria strains is primary recognized by TLR2 and prebiotics like FOS by TLR4 [16, 27]. The GPE used in this study was analyzed for endotoxin pyrogens, and these were within the limits stated by pharmacopeia guidance. However, a recent study showed that pollen is an important source of airborne endotoxin [28]. Therefore, an at least partial effect of LPS as a potential component of the GPE on the observed TLR4 dependence cannot be excluded. Nevertheless, activation or suppression of distinct factors of the TLR4/NFκB and other TLR pathways seems to be crucial for the probiotics-mediated modulation of inflammatory responses and are highly species- and strain-dependent [29]. *Bifidobacterium lactis* is able to suppress NFκB signaling in intestinal epithelial cells [30], and dietary consumption showed enhanced and improved natural immunity [31]. Probiotic mixtures containing *Lactobacillus acidophilus* upregulate the generation of CD4^+^Foxp3^+^ regulatory T cells [32] and increase IL-10 levels [33]. *Lacticaseibacillus rhamnosus* species are well-studied probiotics and are able to enhance tight junction strength [34], reduce NFκB signaling and pro-inflammatory cytokine and chemokine release [35], and show several other immunomodulatory properties [29]. Furthermore, prebiotic supplements like non-digestible short-chain galacto- and long-chain fructo-oligosaccharides have previously showed immunomodulatory effects in the absence of probiotic strains by promoting IL-10 production, upregulation of Foxp3^+^ T cells, and are acting via TLR4 [16]. Interestingly, another study demonstrated that oligosaccharide preparations can be contaminated with LPS, which is responsible for some of the described immunological effects of these preparations [36], an option that cannot be fully ruled out in the current study. Nevertheless, in this ex vivo study, the immunomodulatory potential to downregulate GPE-induced Th2 immune response by combining two probiotic strains with prebiotic FOS, as synbiotic mixes Pollagen® and Kallergen®, could be demonstrated.

To investigate the beneficial mechanisms on GPE-induced cellular immune response a bit more precise, the effects of the synbiotics on MoDCs and subsequent autologous T cell stimulation were analyzed. Upon activation, MoDCs undergo a maturation process and become competent in presenting antigens, which is accompanied by an upregulation of surface maturation markers like CD83, CD86, and HLA-DR. Stimulation with GPE induced comparable changes in maturation marker expression on MoDCs from allergic patients and healthy controls, as demonstrated previously by others [37]. The addition of the synbiotic mixes or synbiotic culture supernatants showed no relevant influence on surface marker expression. Both synbiotic mixes induced raised levels of IL-12p70 as well as increased IFN-γ release by GPE-matured MoDCs. Previous reports confirmed the induction of Th1-like cytokines by *Lactobacillus* and *Bifidobacterium* strains [38–40]. Furthermore, an upregulation of IL-23, which is linked to the generation and maintenance of Th17 cells, as well as of IL-6, a key driver of IL-17 secreting CD4^+^ and CD8^+^ T cells and regulator of T cell proliferation and survival [41], was demonstrated. The mechanisms responsible for Th2 polarization are still not fully understood, but it was shown that early IL-4 production in absence of IL-12 induces Th2 differentiation [42]. In this study, a tendency toward elevated release of Th2 cytokines like IL-4, IL-5, and IL-13 in GPE-stimulated MoDCs from grass pollen–allergic patients compared to healthy controls could be demonstrated. Together, these findings indicate a polarization toward Th1- and Th17-promoting conditions, by adding synbiotics to GPE during maturation of MoDCs. The sole addition of synbiotic culture supernatants did not lead to this significant shift in cytokine secretion, suggesting that the amount or nature of bacterial products released during culturing of the probiotic bacteria was not sufficient to induce the same effects. One limitation of this study, which should be addressed in future analyses, is that the composition and nature of the immunomodulatory metabolites in the synbiotic culture supernatants are not known. Besides short-chain fatty acids that can modulate epithelial integrity and the anti-inflammatory immune response, a variety of other immunomodulatory prebiotic and probiotic metabolites and other secreted molecules have been described. These include secreted protein p40 (barrier function, cell survival, mucin, and IgA production), polysaccharide A (IL-10 production, Treg induction), peptidoglycan (IL-10 production, Treg induction), lipoteichoic acid (barrier function, protective immune response), exopolysaccharides (anti-inflammatory), indole-3-aldehdye (immune responses for epithelial cell protection), and others [43, 44]. Furthermore, both synbiotics were able to induce anti-inflammatory IL-10 release. Both DCs and Tregs are relevant sources of IL-10 [45]. Irrespective of the allergic status of the individuals examined, enhanced IL-10 production by MoDCs matured in the presence of GPE and the synbiotic mixes was observed, which hints to a relevant role of these cells as IL-10 source after contact with the synbiotics. In comparison, IL-10 secretion was very low in supernatants of T cells co-cultured with MoDCs that had matured in the presence of GPE and synbiotic mixes. However, the source of secreted IL-10 in this experimental setup remains elusive. IL-10- and IL-12p70-producing tolerogenic MoDCs are able to induce Treg development [16, 46] and are known to suppress allergen-specific Th2 responses [47]. The balance between the anti-inflammatory cytokine IL-10 and pro-inflammatory cytokine IL-12 is important for host immunity, and the modulation mechanisms by different probiotic *Lactobacillus* and *Bifidobacterium* strains are not yet fully understood [38].

Next, cytokine secretion of co-cultures of matured MoDCs with autologous naïve T cells of grass pollen–allergic patients and healthy controls was analyzed. Th2 cytokine levels (IL-4, IL-5, and IL-13) were significantly higher in supernatants of GPE-matured MoDC-T cell-co-cultures of grass pollen–allergic patients compared to healthy controls. It was demonstrated previously by others that Th2 cytokine production in autologous stimulation assays of allergic patients could be clearly distinguished from healthy individuals [48, 49]. MoDCs matured in the presence of GPE together with the synbiotic mixes, or synbiotic culture supernatants induced a less Th2-like cytokine response in co-culture with naïve T cells. Additionally, the synbiotics increased the levels of IFN-γ, TNF-α, IL-17A, and IL-17F again indicating a shift toward Th1- and Th17-promoting conditions.

Taken together, these results provide evidence that the synbiotic mixes Pollagen® and Kallergen® are efficient in downregulating the GPE-induced Th2 immune response in PBMCs from grass pollen–allergic patients as well as in autologous MoDC-T cell-stimulation assays ex vivo. In addition to elevated IL-10 secretion, the data indicates a shift from a Th2- to a more Th1- and Th17-like phenotype. Although the presented study has limitations, like experimental models for a complex in vivo situation, we still believe that our ex vivo experiments can mimic the immunological events in the mucosa-associated lymphoid tissue (MALT), where intestinal lymphocytes come into contact with synbiotic mixes. MALT structures are the main sources of IgA-producing plasma cells along the surfaces of all mucosal tissues. These structures include M cells, which serve as entry sites for the luminal antigen to induce efficient immune responses [50]. Morphological features of M cells reveal a lack of microvilli but show basolateral pockets, where mononuclear phagocytes and lymphocytes are located in close contact with each other and the M cells and where antigen encounter takes place. The M cells however can be reduced to their role as sheath and particle transfer station. In addition, DCs are constantly sampling the luminal content of the intestine to monitor it for pathogens [51], an interaction that partially is mimicked in the experiments, in which MoDCs matured in the presence of synbiotics were co-cultured with autologous naïve T cells. The present study thereby provides first insights into the immunomodulatory properties of the investigated synbiotic mixes in the context of grass pollen allergy and suggests potential administration of dietary supplements for reduction or even prevention of allergic symptoms.

## Supplementary Information

Below is the link to the electronic supplementary material.

Supplementary file1 (DOCX 7332 KB)

## Data Availability

All data is included in the text; however, the raw data of this article will be made available by the authors, without undue reservation, to any qualified researcher.
