# Supplementary material for: Ex Vivo Immunomodulatory Effects of Lactobacillus-, Lacticaseibacillus-, and Bifidobacterium-Containing Synbiotics on Human Peripheral Blood Mononuclear Cells and Monocyte-Derived Dendritic Cells in the Context of Grass Pollen Allergy
Source: Probiotics Antimicrob Proteins. 2022 Feb 3;15(4):868–79. doi: 10.1007/s12602-022-09920-w (PMC10393851; doi:10.1007/s12602-022-09920-w)

***Ex vivo* immunomodulatory effects of *Lactobacillus*- and *Bifidobacterium*-containing synbiotics on human peripheral blood mononuclear cells and monocyte-derived dendritic cells in the context of grass pollen allergy**

**Probiotics and Antimicrobial Proteins**

Alexander Heldner, Matthew D. Heath, Benjamin Schnautz, Sebastian Kotz, Adam Chaker, Matthias F. Kramer, Constanze A. Jakwerth, Ulrich M. Zissler, Carsten B. Schmidt-Weber, Simon Blank

**Corresponding author:**

Simon Blank

Center of Allergy and Environment (ZAUM)

Technical University of Munich and Helmholtz Center Munich

Email: simon.blank@tum.de

**Supplemental Fig. 1. Gating strategy for FACS analysis of Gata3^+^ cells**

The FlowJo_V10 software (FlowJo LLC, Ashland, Oregon, USA) was used to select Gata3^+^ cells on 2-D plots. After excluding cell debris and gating on single cells, living CD45^+^ leukocytes were selected. Lymphocytes were then gated on CD3^+^/CD4^+^ cells. Among that double positive T cell population, proliferating Gata3^+^ cells were selected and changes in transcription factor expression further analyzed. Shown are four representative gatings for PBMCs from a grass pollen-allergic patient, which were untreated or stimulated with grass pollen extract (GPE) w/o synbiotic mixes.


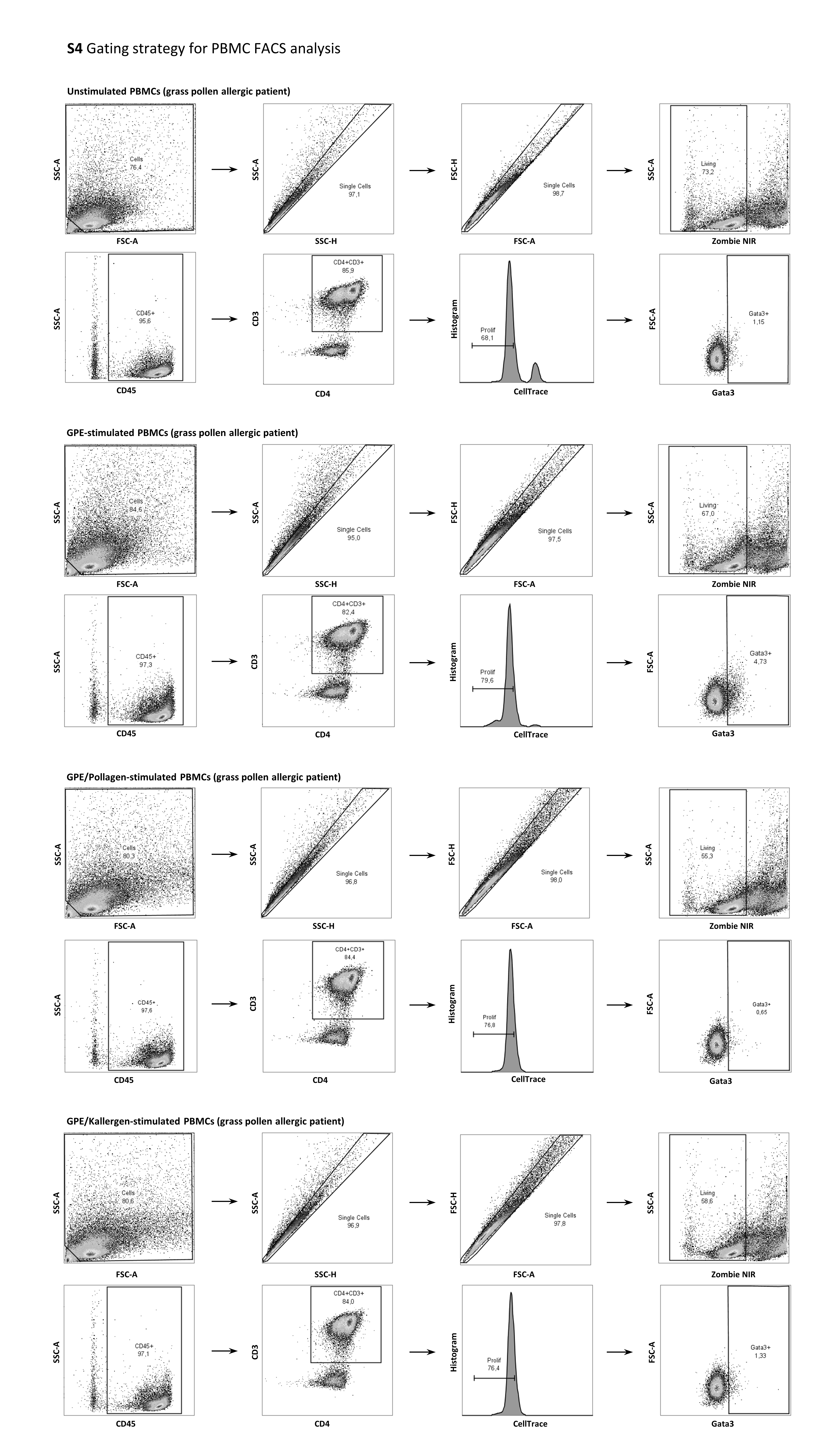


**Supplemental Fig. 2. Stimulation of PBMCs with phytohaemagglutinin**

PBMCs from grass pollen allergic patients (black squares) and healthy controls (white squares) were stimulated with phytohaemagglutinin (PHA) for seven days. PHA served as positive control. Supernatants were analyzed for the levels of IL-4, IL-5, IL-9, IL-13, IL-10 and IFN-γ. A dotted line indicates the upper detection limit for IFN-γ. Results are presented as mean ± SD.

**
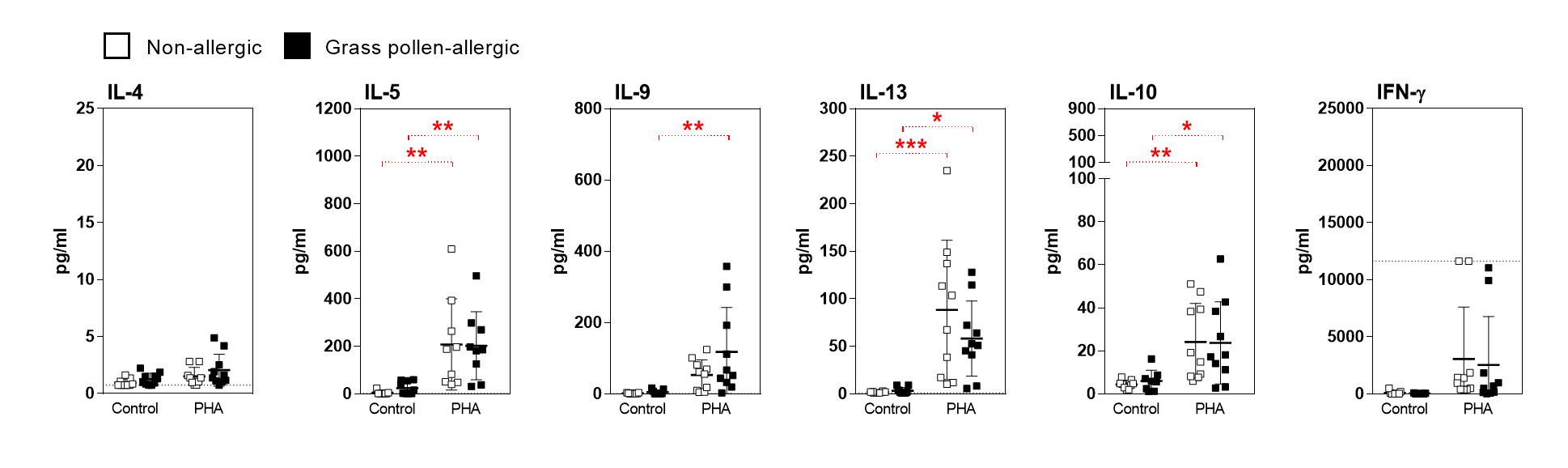
**

**Supplemental Fig. 3. Downregulation of GPE-driven Th17 cytokine production in grass pollen-allergic patients’ PBMCs by synbiotic mixes**

PBMCs from grass pollen-allergic patients (black squares) and healthy controls (white squares) were stimulated with grass pollen extract (GPE) for seven days. Stimulation was done w/o Pollagen (Pol) / Kallergen (Kal), LPS-RS Ultrapure (aTLR4) and Pollagen / Kallergen supernatant (Pol S / Kal S). Phytohaemagglutinin (PHA) served as positive control. Supernatants were analyzed for the levels of IL-17A and IL17F. Results are presented as mean ± SD. Significant differences between controls and Pollagen, controls and Kallergen and within controls are indicated in black, blue and red, respectively.


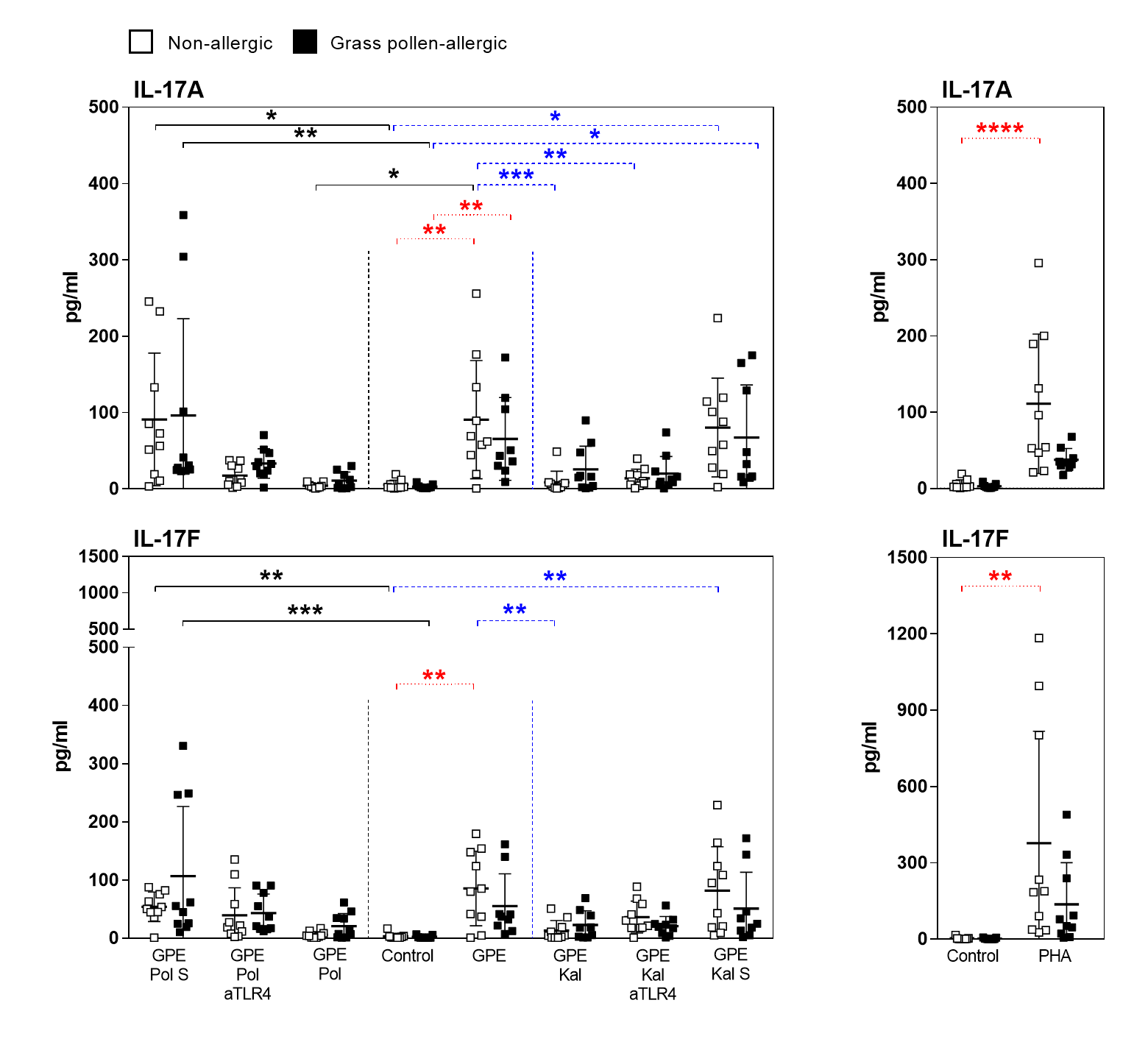


**Supplemental Fig. 4. Partially blocking of Pollagen-mediated downregulation of GPE-induced Gata3 expression**

PBMCs from grass pollen allergic patients (black squares) and healthy controls (white squares) were stimulated with grass pollen extract (GPE) for seven days. Stimulation was done w/o Pollagen (Pol) and PAb-hTLR2 (aTLR2). Gata3^+^ cells of proliferating CD3^+^/CD4^+^ cells were analyzed by FACS. Results are presented as mean ± SD.


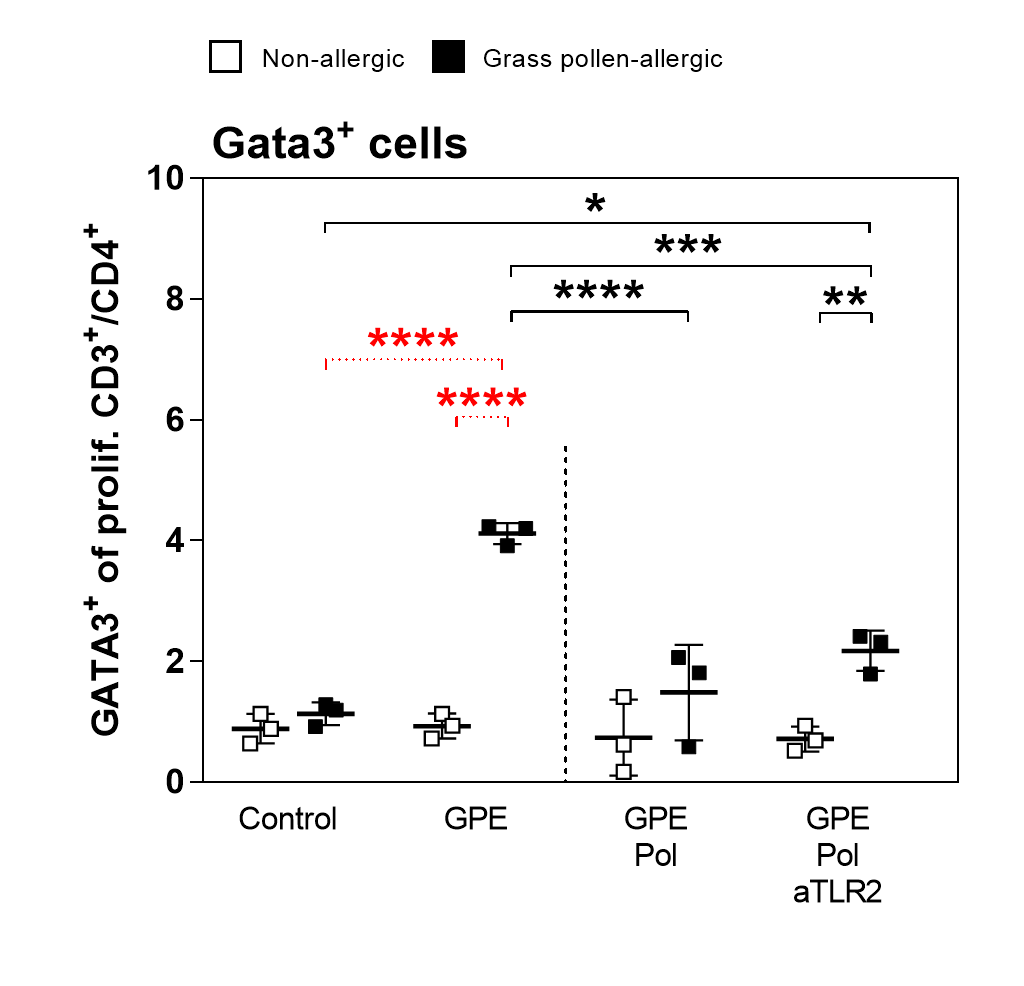


**Supplemental Fig. 5. GPE-induced FoxP3 expression in PBMCs is not altered by synbiotic mixes**.

PBMCs from grass pollen allergic patients (n=10; black squares) and healthy controls (n=10; white squares) were stimulated with grass pollen extract (GPE) for seven days. Stimulation was done w/o Pollagen (Pol) / Kallergen (Kal), LPS-RS Ultrapure (aTLR4) and Pollagen / Kallergen supernatant (Pol S / Kal S). Phytohaemagglutinin (PHA) served as positive control. FoxP3^+^ cells of proliferating CD3^+^/CD4^+^ cells were analyzed by FACS. For each patient, FoxP3 expression in the negative control was set as 1 and fold change to control was calculated for each stimulation condition. Results are presented as mean ± SD. Significant differences between controls and Pollagen, controls and Kallergen and within controls are indicated in black, blue and red, respectively.

**
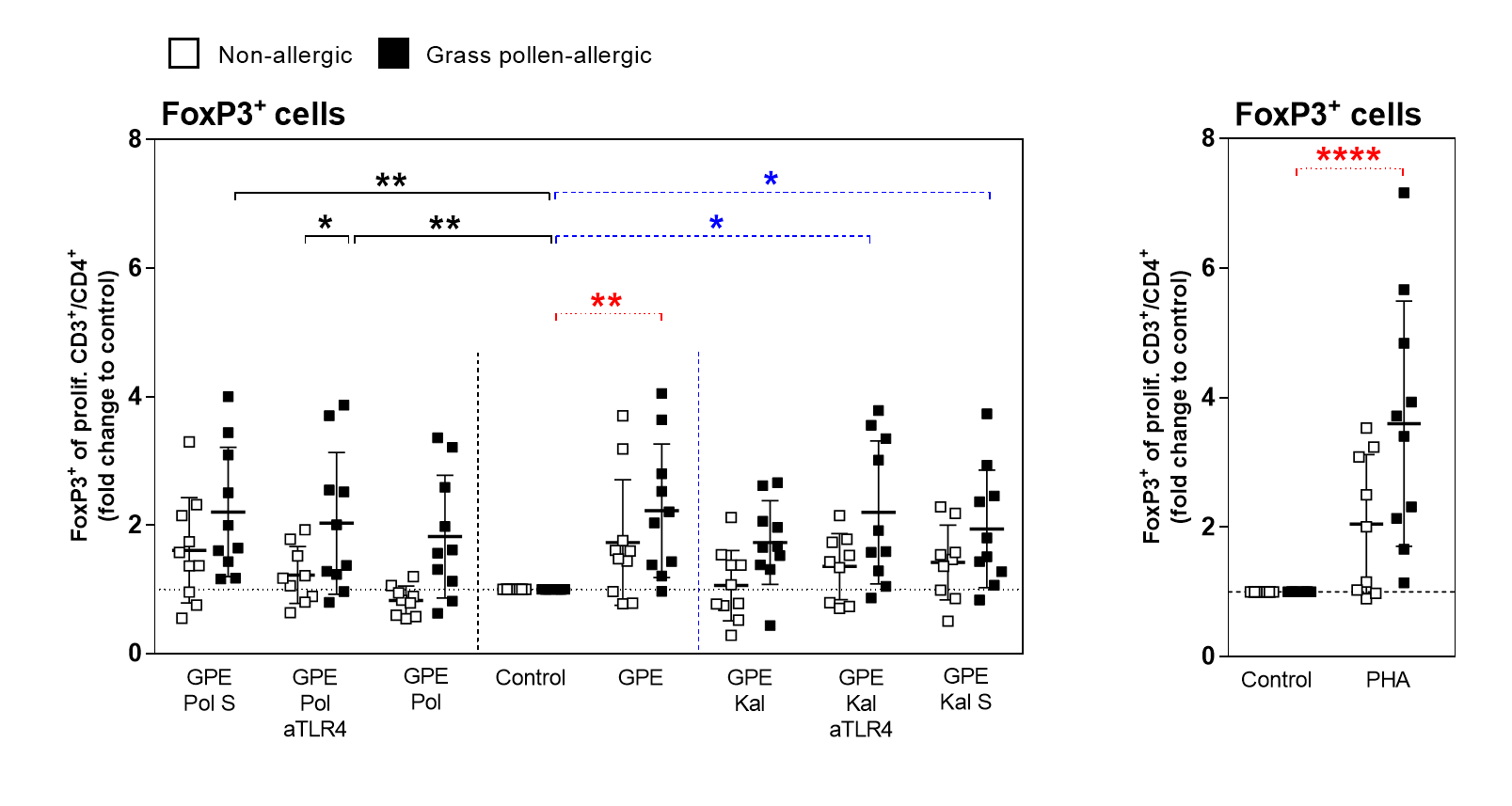
**

**Supplemental Fig. 6. Regulation of GPE-driven MoDC maturation by synbiotic mixes**

Immature MoDCs from grass-pollen allergic patients (black bars) and healthy controls (white bars) were stimulated with grass pollen extract (GPE) for seven days. Stimulation was done w/o Pollagen (Pol) / Kallergen (Kal) and Pollagen / Kallergen supernatant (Pol S / Kal S) and mean fluorescence intensity (∆MFI) of MoDC surface maturation markers CD83, CD86, HLA-DR, CD80 and CD209 was measured. Lipopolysaccharide (LPS) and lipoteichoic acid (LTA) served as positive controls. Results are presented as mean ± SD. Significant differences between controls and Pollagen, controls and Kallergen and within controls are indicated in black, blue and red, respectively.


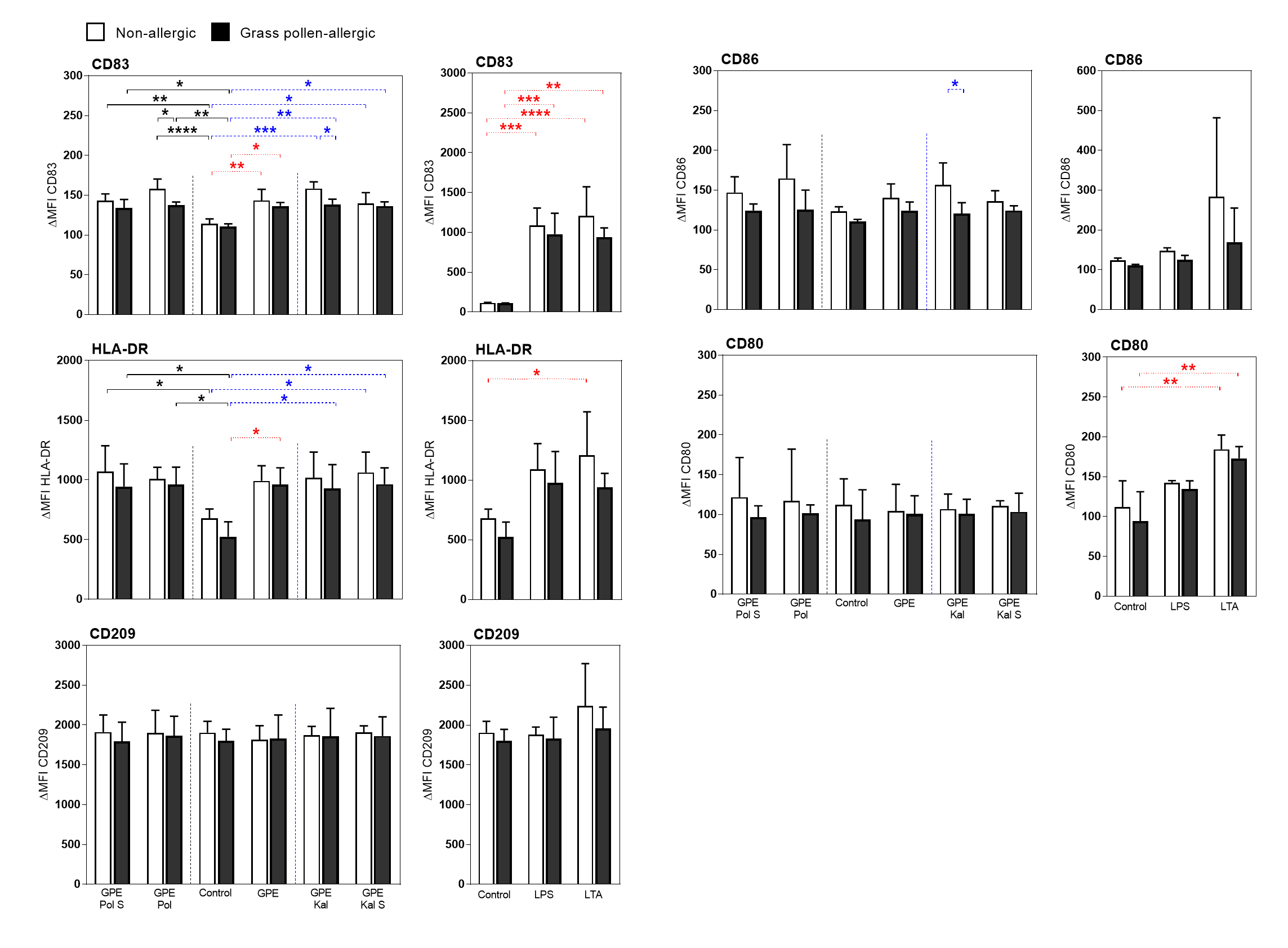


**Supplemental Fig. 7. Cytokine secretion of matured MoDCs**

Immature MoDCs from grass-pollen allergic patients (black squares) and healthy controls (white squares) were stimulated with grass pollen extract (GPE) for 24 hours. Stimulation was done w/o Pollagen (Pol) / Kallergen (Kal) and Pollagen / Kallergen supernatant (Pol S / Kal S). Lipopolysaccharide (LPS) and lipoteichoic acid (LTA) served as positive controls. Supernatants were analyzed for the levels of IL-5, IL-13 and IFN-γ. Results are presented as mean ± SD.


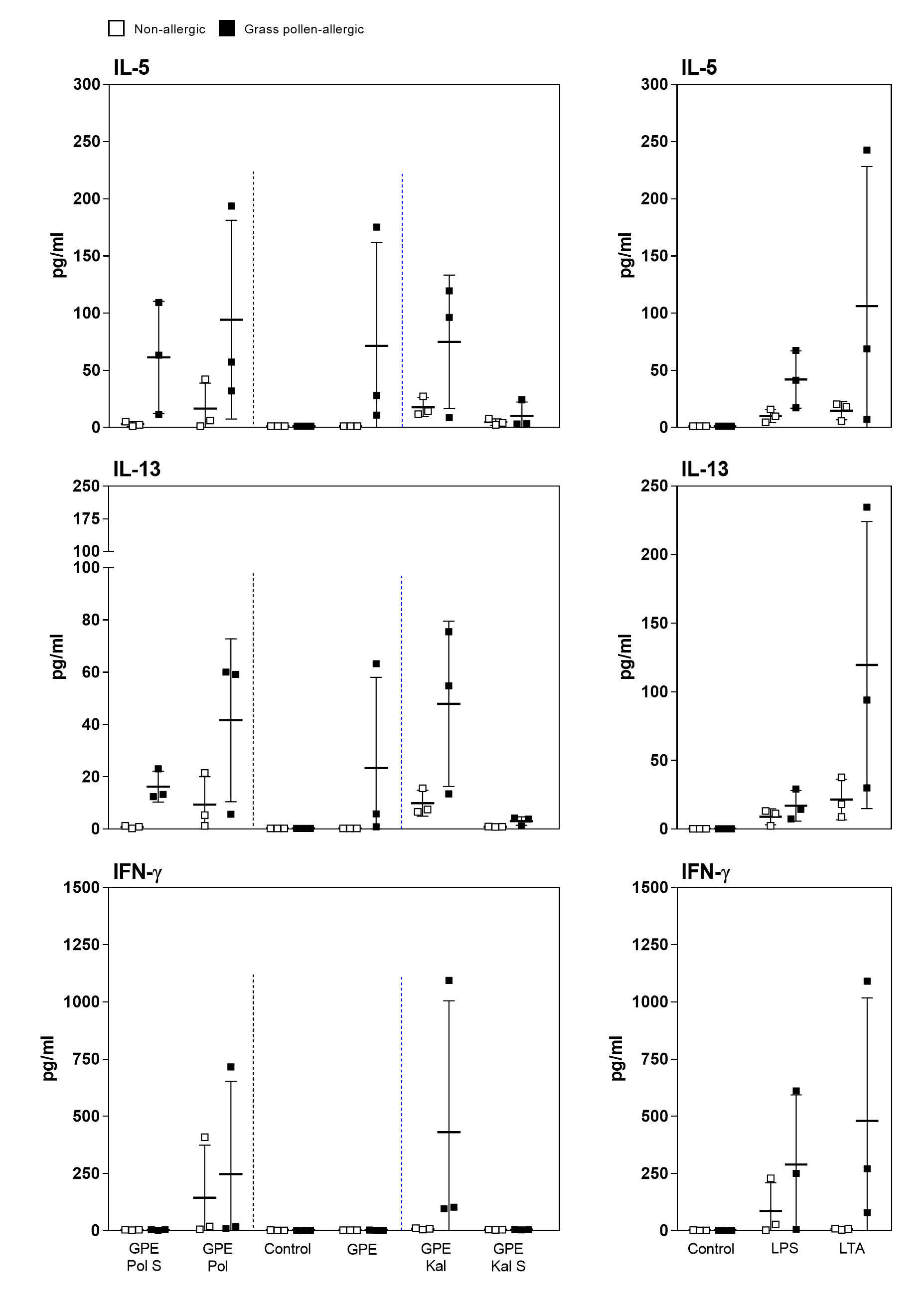


**Supplemental Fig. 8. Cytokines in supernatants of autogenic co-cultures of naïve T cells and LPS- or LTA-matured MoDCs**

Autogenic naïve T cells from grass-pollen allergic patients (black squares) and healthy controls (white squares) were co-cultured with lipopolysaccharide (LPS)- or lipoteichoic acid (LTA)-matured MoDCs from the same donors. Supernatants were analyzed for the levels of IL-4, IL-5, IL-9, IL-10, IL-13, IL-17A, IL-17F, TNF-α and IFN-γ. Results are presented as mean ± SD.


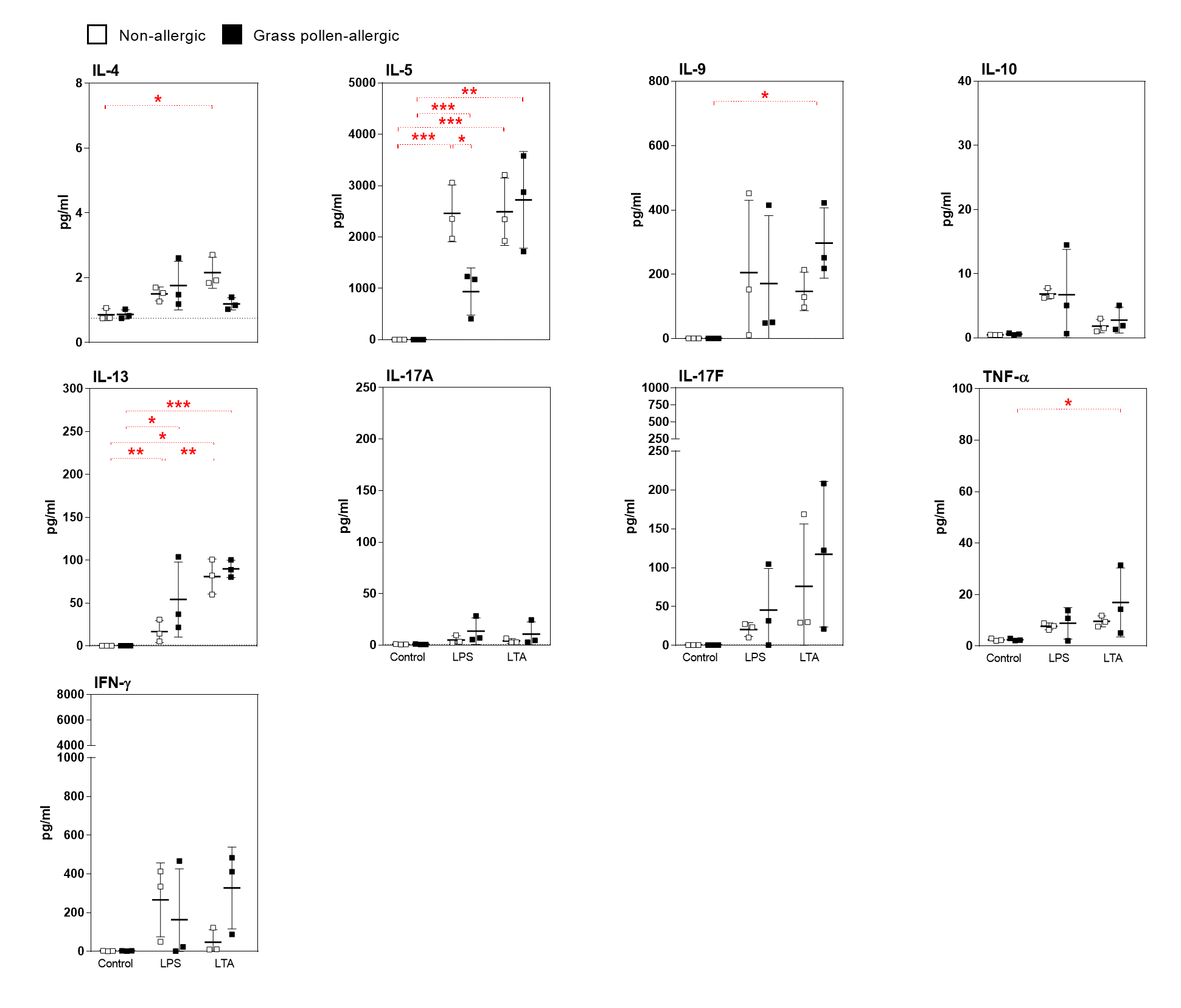

Supplement: Supplementary file 1 — Supplementary file1 (DOCX 7332 KB) [file 12602_2022_9920_MOESM1_ESM.docx]
